# A Combined Multi-Cohort Approach Reveals Novel and Known Genome-Wide Selection Signatures for Wool Traits in Merino and Merino-Derived Sheep Breeds

**DOI:** 10.3389/fgene.2019.01025

**Published:** 2019-10-25

**Authors:** Sami Megdiche, Salvatore Mastrangelo, Mohamed Ben Hamouda, Johannes A. Lenstra, Elena Ciani

**Affiliations:** ^1^Départment des Ressources Animales, Agroalimentaire et Développement Rural, Institut Supérieur Agronomique de Chott-Mariem, Université de Sousse, Sousse, Tunisia; ^2^Dipartimento di Bioscienze, Biotecnologie e Biofarmaceutica, University of Bari “Aldo Moro,” Bari, Italy; ^3^Dipartimento di Scienze Agrarie, Alimentari e Forestali, University of Palermo, Palermo, Italy; ^4^INRA-Tunisie, Institute for Risk Assessment Sciences, Ariana, Tunisia; ^5^Faculty of Veterinary Medicine, Utrecht University, Utrecht, Netherlands

**Keywords:** Merino sheep breeds, wool, genome-wide selection signatures, F_ST_-outlier, local ancestry in admixed populations, runs of homozygosity

## Abstract

Merino sheep represents a valuable genetic resource worldwide. In this study, we investigated selection signatures in Merino (and Merino-derived) sheep breeds using genome-wide SNP data and two different approaches: a classical F_ST_-outlier method and an approach based on the analysis of local ancestry in admixed populations. In order to capture the most reliable signals, we adopted a combined, multi-cohort approach. In particular, scenarios involving four Merino breeds (Spanish Merino, Australian Merino, Chinese Merino, and Sopravissana) were tested *via* the local ancestry approach, while nine pair-wise breed comparisons contrasting the above breeds, as well as the Gentile di Puglia breed, with non-Merino breeds from the same geographic area were tested *via* the F_ST_-outlier method. Signals observed using both methods were compared with genome-wide patterns of distribution of runs of homozygosity (ROH) islands. Novel and known selection signatures were detected. The most reliable signals were observed on OAR 3 (*MSRB3* and *LEMD3*), OAR10 (*FRY* and *RXFP2*), OAR 13 (*RALY*), OAR17 (*FAM101A*), and OAR18 (*NFKBIA, SEC23A*, and *PAX9*). All the above overlapped with known QTLs for wool traits, and evidences from the literature of their involvement in skin/hair/wool biology, as well as gene network analysis, further corroborated these results. The signal on OAR10 also contains well known evidence for association with horn morphology and polledness. More elusive biological evidences of association with the Merino phenotype were observed for a number of other genes, notably *LOC101120019* and *TMEM132B* (OAR17), *LOC105609948* (OAR3), *LOC101110773* (OAR10), and *EIF2S2* (OAR17). Taken together, the above results further contribute to decipher the genetic basis underlying the Merino phenotype.

## Introduction

Sheep were among the first livestock species to be domesticated (Ryder 1981). Archeological evidences suggest domestication occurred in a region extending from the northern Zagros to southeastern Anatolia ca. 11,000 B.P. (Zeder, 2008). In the last two decades, information from molecular data, as well as discovery and study of novel archaeological sites, has shed new light on the origins and subsequent diffusion of domestic sheep worldwide (Chessa et al., 2009; Meadows et al., 2011; Kijas et al., 2012; Demirci et al., 2013; Singh et al., 2013; Dymova et al., 2017; Ethier et al., 2017; Ivanova et al., 2018a; Ivanova et al., 2018b). Early domesticated sheep are known to have been transported over long distances or even by sea, as early as around 12,000 years BP (Zeder, 2008). They are supposed to have been initially reared mainly for meat and, only during the fifth millennium B.P., specialization for “secondary” products, such as milk and wool, is thought to have occurred (Debono Spiteri et al., 2016). In particular, analysis of viral retro-types combined with archaeological evidence provide support to the hypothesis that specialized wool sheep populations were developed in South-West Asia and then spread throughout Europe, replacing, in the majority of areas, the more primitive domestic stocks (Chessa et al., 2009). Specialization for wool production culminated, in the Middle Ages, with the development of the Merino sheep in Spain. In a recent paper, by analyzing genome-wide SNP data from an intercontinental set of sheep breeds, inclusive of 12 Merino and Merino-derived populations, our group contributed to the reconstruction of the history of Merino development, and the subsequent worldwide merinization process (Ciani et al., 2015).

Well renowned for its premium white fleece and the abundant production of soft, fine, and curly wool, Merino sheep represent a valuable genetic resource worldwide. As such, deciphering the genetic basis underlying the peculiar Merino phenotype is a fundamental aim, and it may further contribute improving wool performances of Merino and Merino-derived breeds. A number of papers have addressed this issue, looking at the genome in search for QTLs (quantitative trait loci) related to wool traits, by using STR markers in Merino (Beh et al., 2001; Bidinost et al., 2008; Roldan et al., 2010), Merino crosses (Rogers et al., 1994; Henry et al., 1998;Zhai et al., 2019), and non-Merino (Allain et al., 1998; Ponz et al., 2001; Allain et al., 2006) sheep populations, or looking at candidate genes (Ling et al., 2014; Rong et al., 2015; Ma et al., 2017; Mu et al., 2017), with keratin genes being among the most studied targets (Parsons et al., 1994; Phuaa et al., 2015; Chai et al., 2017; Li et al., 2017a; Li et al., 2017b; Sulayman et al., 2018; Gong et al., 2019). With the advent of SNP array genotyping technologies, genome-wide association studies (GWAS) using bi-allelic markers have become feasible, and they have been performed in the ovine species to investigate, among others, wool traits (Wang et al., 2014; Bolormaa et al., 2017). An additional approach for connecting DNA to phenotype is the detection of evidence of selective pressure in specific genomic regions by using genome-wide SNP genotype data, also referred to as “selection signatures” analysis. This method has emerged mainly because (i) it does not require the use of phenotypic records, and (ii) unlike GWAS, it can detect selection signatures also when anthropogenic selection has determined complete fixation of the favorable allele (e.g., Qanbari et al., 2014). These features are both relevant in studies addressing genotype–phenotype associations for wool traits, where availability of phenotypic records may represent a limiting issue, and long-term intensive human selection toward wool attributes is likely to have been responsible for the complete prevalence, in the selected populations, of the desired allele. In some of the studies where selection signatures for wool traits have been described, identification of regions affecting wool attributes was not the unique or major goal, with repercussions of this conceptual set-up on the choice of breeds to be contrasted (Zhang et al., 2013; Fariello et al., 2014; Wang et al., 2015; Wei et al., 2015; Seroussi et al., 2017; Rochus et al., 2018). To our knowledge, only two analyses of selection signatures specifically targeting wool attributes have been performed so far (Demars et al., 2017; Gutierrez-Gil et al., 2017). Out of them, only the latter was centered on Merino sheep, which were contrasted, in that study, to the coarse-wool Churra sheep from Spain. Our study follows up on the work by Gutierrez-Gil et al. (2017) to further investigate selection signatures in various Merino (and Merino-derived) sheep breeds under different scenarios, using two different approaches: a classical F_ST_-outlier method and a less usual one based on the analysis of local ancestry in admixed populations (Sankararaman et al., 2008). Signals observed using both methods are also compared with genome-wide patterns of distribution of ROH (runs of homozygosity) islands. We specifically adopted here a multi-cohort approach with the aim of retaining only the most reliable signals.

## Materials and Methods

### Genotypic Data

A total of 459 unrelated animals arranged in 11 breeds were used in this study (**Table S1**). Out of them, six were Merino or Merino-derived breeds (Spanish Merino, Australian Merino, Rambouillet, Gentile di Puglia, Sopravissana, and Chinese Merino), and five had no known Merino background (Churra, Ojalada, Bergamasca, Appenninica, and Tibetan) and belonged to the category of “coarse wool” sheep, not purposely selected for wool quality traits (**Data Sheet 1**). SNP genotypes had been generated in previous published studies (**Table S1**) by using the Illumina OvineSNP50 Genotyping BeadChip. The whole SNP genotype dataset is available on the WIDDE database (http://widde.toulouse.inra.fr/widde/). The following quality control criteria were applied: (i) individuals with genotyping rate ≤ 90% (command *–mind 0.1*) were removed; (ii) loci with call rate ≤ 99% (command *–geno 0.01*), minor allele frequency ≤ 0.005 (command *–maf 0.005*), and non-autosomal loci were removed; and SNP positions were updated according to the sheep map version Oar_V4. All the above procedures were performed using the PLINK software v. 1.07 (Purcell et al., 2007).

### Inference of Local and Global Merino Ancestry

We used the LAMP (Local Ancestry in adMixed Populations) software (Sankararaman et al., 2008) to estimate the individual’s local ancestry of Merino proportion under various scenarios, each contrasting a Merino *versus* a non-Merino breed, for a total of four cohorts (**Table S2**). LAMP is a method for estimating ancestries at each locus in a population of admixed individuals i.e., populations formed by the mixing of two or more ancestral populations. The software operates on sliding windows of contiguous SNPs and assigns ancestries by combining the results with a majority vote. The following default settings were adopted: number of generations since admixture (g) = 7 and recombination rate (r) = 1E−08. We opted for adopting default settings in all the tested scenarios since (i) the method was shown to provide robust estimates under different setting configurations in both the literature (Sankararaman et al., 2008) and our preliminary analyses (data not shown). The fraction of global admixture (α) was determined, for each scenario, using the ADMIXTURE software (Alexander et al., 2009). We ran LAMP in the LAMPANC mode, i.e., providing allele frequencies of the two ancestral population proxies. The LAMP analysis provides, among other output results, the marker average ancestry (MAA) related to the two considered ancestral populations. Only MAAs representing the Merino fraction were considered in this study to identify the significant region supposed to be under selection. To this aim, both of the following criteria should be respected by the putative selection signature: (i) local Merino MAA higher than the genome-wide Merino MAA and (ii) being included in the top 5% of SNPs ranked by MAA of Merino proportion.

### Detection of F_ST_-Outlier Markers

We adopted the F_ST_-outlier approach implemented in BayeScan (Foll and Gaggiotti, 2008) to detect markers putatively under differential selection pressure in Merino and non-Merino sheep breeds, respectively. To this aim, we performed nine pair-wise comparisons, contrasting each time a Merino *versus* a non-Merino sheep breed (**Table S3**). For each cohort, loci that displayed q-val < 0.05 were retained as putatively under selection. Next, we looked for loci that resulted to be putatively under selection in at least four pair-wise comparisons out of nine. For each SNP satisfying the above criteria, we then moved upstream and downstream its position, looking for additional loci with q-val < 0.05 in at least a single pair-wise comparison, and located within 200 kb intervals. We repeated the above process until the next SNP with q-val < 0.05 in at least a single pair-wise comparison was located at a distance higher than 200 kb. Finally, we defined the regions putatively under selection based on the position of the first and the last of the SNPs satisfying the above criteria.

### Runs of Homozygosity

ROH were estimated for each animal belonging to the considered breeds using PLINK (Purcell et al., 2007). The following criteria were used to define the ROH: (i) no missing SNP and no heterozygous genotype were allowed in the ROH, (ii) the minimum number of SNPs that constituted the ROH was set to 25, (iii) the minimum SNP density per ROH was set to one SNP every 100 kb, and (iv) the maximum gap between consecutive homozygous SNPs was 250 kb. The minimum length that constituted the ROH was set to 500 Mb. To identify the genomic regions of high homozygosity, the amount of times that each SNP appeared in the ROH was considered and normalized by dividing it by the number of animals included in the analysis. To identify the genomic regions of “high homozygosity,” also called ROH islands, the top 0.999 SNPs of the percentile distribution of the locus homozygosity range within each breed were selected.

### Gene and QTL Content of Regions Identified as Under Selection

Annotated genes within the genomic regions putatively under selection were obtained from https://www.ncbi.nlm.nih.gov/genome/gdv/browser/?context=gene&amp;acc=101104604 (NCBI Sheep Genome Data Viewer). The Sheep QTL Database, available at https://www.animalgenome.org/cgi-bin/QTLdb/OA/srchloc?chrom=19&qrange=454178-607539&submit=GO, was interrogated for the presence of QTLs (quantitative trait loci) and significant association signals in the genomic regions identified in this study as putatively under selection. To investigate the biological function and the phenotypes that are known to be affected by each annotated gene, we conducted a comprehensive search in the available literature and public databases, such as NCBI (https://www.ncbi.nlm.nih.gov/), GeneCards (https://www.genecards.org), UniProt (www.uniprot.org), and Amigo2 Gene Ontology database (http://amigo.geneontology.org/amigo). Furthermore, we performed a gene network analysis by using GeneMANIA (Mostafavi et al., 2008). This tool allows to build weighted interaction networks using as a source a very large set of functional association data including protein and genetic interactions, pathways, co-expression, co-localization, and protein domain similarity (see http://pages.genemania.org/help/for a more detailed description of the considered network categories).

## Results

### Signals of Selection Detected *via* “Local Ancestry”

Preliminary to the local ancestry analysis, we performed a “global ancestry” analysis using the Bayesian approach implemented in the software ADMIXTURE (Alexander et al., 2009). Individual proportions of global admixture (α) are presented, for the four considered breeds, in **Figure S1**. The observed patterns support, for all the tested breeds, the formulated scenarios, i.e., that each breed could be considered to derive from the crossbreeding of a given Merino and a given non-Merino breed (breed A and breed B, respectively, in **Table S2**). Putatively selected regions, identified from LAMP results, are shown, for the four considered breeds, in **Table S4**–**S7**. An excess of Merino ancestry was observed at 26, 24, 17, and 22 regions for Australian Merino, Chinese Merino, Sopravissana, and Spanish Merino, respectively. A number of regions were shared by at least three out of the four breeds (Table 1) and, among them, two large regions, on OAR 17 (overlapping signals at 48,474,658–58,410,640 bp) and OAR 18 (overlapping signals at 42,864,163–51,943,741 bp), were shared by all the four breeds.

**Table 1:** Signals of selection detected *via* local ancestry, shared by at least three of the four considered breeds.

OAR	Australian Merino				Chinese Merino				Sopravissana				Spanish Merino			
		SNP ID	Position (bp)	MAA		SNP ID	Position (bp)	MAA		SNP ID	Position (bp)	MAA		SNP ID	Position (bp)	MAA
5	Start	rs418698529	26341551	1	Start	rs414589041	56778930	0.97	Start	rs409779782	34321292	0.89				
	End	rs414589041	56778930	1	End	rs415945949	66146282	0.97	End	rs407964514	56019746	0.89				
13	Start	rs421927509	62007588	1	Start	rs160614980	67099575	0.97					Start	rs160604475	63340057	0.92
	End	rs421743434	82928768	1	End	rs421743434	82928768	0.97					End	rs421743434	82928768	0.92
14	Start	rs405740814	173039	1	Start	rs403113459	6678882	0.97					Start	rs405740814	173039	0.80
	End	rs409789065	18615310	1	End	rs423800989	12841601	0.97					End	rs407274307	22608758	0.80
15	Start	rs398486856	747553	0.97					Start	rs430560325	24611939	0.87	Start	rs398442436	19097698	0.96
	End	rs417609233	48071326	0.97					End	rs410686010	42463484	0.87	End	rs404810360	39888859	0.96
16	Start	rs429048373	41142879	0.93					Start	rs419085314	41237027	0.91	Start	rs403615656	31260763	0.92
	End	rs399764897	49217439	0.93					End	rs421054947	55127918	0.91	End	rs408523982	54928813	0.92
*17*	*Start*	*rs398968259*	*20314798*	*0.97*	*Start*	*rs424790285*	*47829307*	*0.95*	*Start*	*rs419855399*	*46130137*	*0.91*	*Start*	*rs426738329*	*48474658*	*0.88*
	*End*	*rs414015395*	*71582708*	*0.97*	*End*	*rs400781870*	*72084885*	*0.95*	*End*	*rs411845108*	*58410640*	*0.91*	*End*	*rs414015395*	*71582708*	*0.88*
*18*	*Start*	*rs416601769*	*42864163*	*0.97*	*Start*	*rs414805156*	*33332554*	*1*	*Start*	*rs401597794*	*28792875*	*0.83*	*Start*	*rs415215918*	*42698731*	*0.88*
	*End*	*rs416850669*	*51943741*	*0.97*	*End*	*rs400436533*	*59172019*	*1*	*End*	*rs415998758*	*68381958*	*0.83*	*End*	*rs415998758*	*68381958*	*0.88*
19	Start	rs409364012	28430094	0.97	Start	rs419333175	31614145	0.97					Start	rs408609148	34392185	0.84
	End	rs412219091	53122560	0.97	End	rs412185520	60327629	0.97					End	rs412185520	60327629	0.84

OAR, sheep chromosome. SNP ID, name of the SNP locus. MAA, marker average ancestry (see the section Materials and methods). In italics, regions that were shared by all the four breeds.

### Signals of Selection Detected *via* the “F_ST_-Outlier” Method

Results of the analyses performed using the F_ST_-outlier approach implemented in BayeScan are presented, for the nine pair-wise comparisons involving Merino and non-Merino breeds, in **Table S8**. The highest number of significant SNPs (277) was detected for the contrast Chinese Merino *vs*. Tibetan. A summary of the obtained results is presented in Table 2. Four regions putatively under differential selection pressure were identified, on OAR3 (15,382,6281–154,318,689 bp), OAR10 (29,392,142–29,776,019 bp), OAR13 (62,707,138–62,747,155 bp), and OAR19 (454,178–607,539 bp). Interestingly, in the region on OAR13, one SNP (rs415003205) had q-val < 0.05 in all the nine considered pair-wise comparisons. This is a deep intronic variant (G/A) located at 5,264 bp downstream the end of the first exon of the RALY gene. For the region on OAR3, the locus displaying the highest number of pair-wise comparisons showing signal of selection (six out of nine) was rs423370130 (154,072,493 bp). For the region on OAR19, the highest number of pair-wise comparisons showing signal of selection was five (rs404730996). The large region on OAR10 displayed a maximum of six pair-wise comparisons showing signal of selection, at 29,413,536 bp (rs401979890). In the same region, two loci, out of which one (rs414794714, at 29776019 bp) rather far from rs401979890, had five pair-wise comparisons showing signal of selection. This pattern suggests that the considered region may harbor two different selection signatures.

**Table 2:** Summary results of the F_ST_-outlier approach for the nine pair-wise comparisons between Merino and non-Merino breeds.

Loci			Pair-wise comparisons									N
OAR	SNP ID	Position	1	2	3	4	5	6	7	8	9	
3	rs429917763	153826281										2
3	rs426111530	153889169										2
3	rs408016275	153927239										3
3	rs414901427	153976304										3
3	rs409568101	153996225										1
3	rs416115321	154033734										2
3	rs423370130	154072493										6
3	rs417916710	154223123										2
3	rs159858948	154318689										3

10	rs419203432	29392142										3
10	rs401979890	29413536										6
10	rs413264476	29453722										1
10	rs424871667	29479711										5
10	rs399348601	29489616										3
10	rs425859016	29660838										1
10	rs404720287	29685665										2
10	rs415997827	29742016										2
10	rs414794714	29776019										5

13	rs401457425	62707138										4
13	rs415003205	62747155										9

19	rs421064536	454178										1
19	rs409839516	504608										2
19	rs404730996	566456										5
19	rs424406294	607539										1

OAR, sheep chromosome. SNP ID, name of the SNP locus. N, number of pair-wise comparisons showing signal of selection for the considered SNP. 1, Australian Merino vs. Churra. 2, Australian Merino vs. Ojalada. 3, Spanish Merino vs. Churra. 4, Spanish Merino vs. Ojalada. 5, Gentile di Puglia vs. Appenninica. 6, Gentile di Puglia vs. Bergamasca. 7, Sopravissana vs. Appenninica. 8, Sopravissana vs. Bergamasca. 9, Chinese Merino vs. Tibetan.

The loci that provided overlapping signals for the same Merino (or Merino-derived) breed with both the “local ancestry” and the “F_ST_-outlier” methods are highlighted in **Table S8**, while in **Tables S4** to **S7**, the putatively selected regions, detected using the “local ancestry” method, where at least one significant SNP in at least one pair-wise comparison of the “F_ST_-outlier” method involving the corresponding Merino (or Merino-derived) breed was observed, are highlighted in light yellow. In general, the majority of the regions (16/26, Australian Merino; 15/24, Chinese Merino; 9/17 Sopravissana; 12/22 Spanish Merino) showed overlapping signals between the two methods.

### Gene and QTL Content of Putatively Selected Regions

The two large regions on OAR 17 and 18, detected as putatively selected by “local ancestry” analysis, contain 148 and 70 genes, respectively (**Table S9** and **S10**). These regions were screened for the presence of known QTLs in sheep (**Table S11A**). Interestingly, we found two QTLs associated with a wool trait, notably “greasy fleece weight,” at positions 49,606,819–49,606,859 bp and 51,061,367–51,061,407 bp, respectively, in OAR17 (Ebrahimi et al., 2017), and one QTL associated with “staple length,” at position 9,943,363–68,604,602 bp in OAR18 (Allain et al., 2006). The OAR17 QTL at position 49,606,819–49,606,859 bp is located in an inter-genic region, between *LOC101120019* (60S ribosomal protein L10a-like, at position 49,486,725–49,520,271 bp) and *TMEM132B* (transmembrane protein 132B, at position 49,844,529–50,246,808 bp) (data not shown). Similarly, the OAR17 QTL at position 51,061,367–51,061,407 bp is located in an inter-genic region, between *LOC101115905* (refilin-A, alias “family with sequence similarity 101, member A” or “filamin-interacting protein FAM101A,” at position 51,021,418–51,035,210 bp) and *LOC106991703* (a long non-coding RNA, at position 5,118,8762–51,255,313 bp) (data not shown). The large OAR18 QTL at position 9,943,363–68,604,602 bp includes 693 genes (data not shown) and was hence not useful to refine the signal position. Therefore, the 70 genes detected in the putatively selected region on OAR18 were screened for inclusion in the output of the human GeneCards database using the queries “hair,” “wool,” and “horn,” selected as the most representative of the Merino phenotype. While none of the genes was retrieved when using “wool” or “horn” keywords, a total of 16 out of 70 (23%) genes were retrieved when using the keyword “hair” (**Table S10**). Notably, three genes displayed particularly high GeneCards relevance scores: *NFKBIA* (16.1), *SEC23A* (15.27), and *PAX9* (7.17).

The four regions detected as putatively selected by “F_ST_-outlier” analysis contain five (OAR3), four (OAR10), one (OAR13), and two (OAR19) genes (**Tables S12A–D**). Also, these regions were screened for the presence of known QTLs in sheep (**Table S11B**). On OAR3, a QTL associated with wool traits (notably, “staple length”) had been previously mapped, within a large chromosome interval (region 1,184,337–224,283,230 bp) encompassing the region detected in this study (Ponz et al., 2001). On OAR10, a genome-wide association study for wool traits in Chinese Merino detected a significant SNP for fiber diameter at position 30 Mb, and several SNPs significant for crimp at 26–27 Mb (Wang et al., 2014). On OAR13, one SNP at 62.9 Mb was associated with wool fiber diameter (Bolormaa et al., 2017).

**Figure 1:**
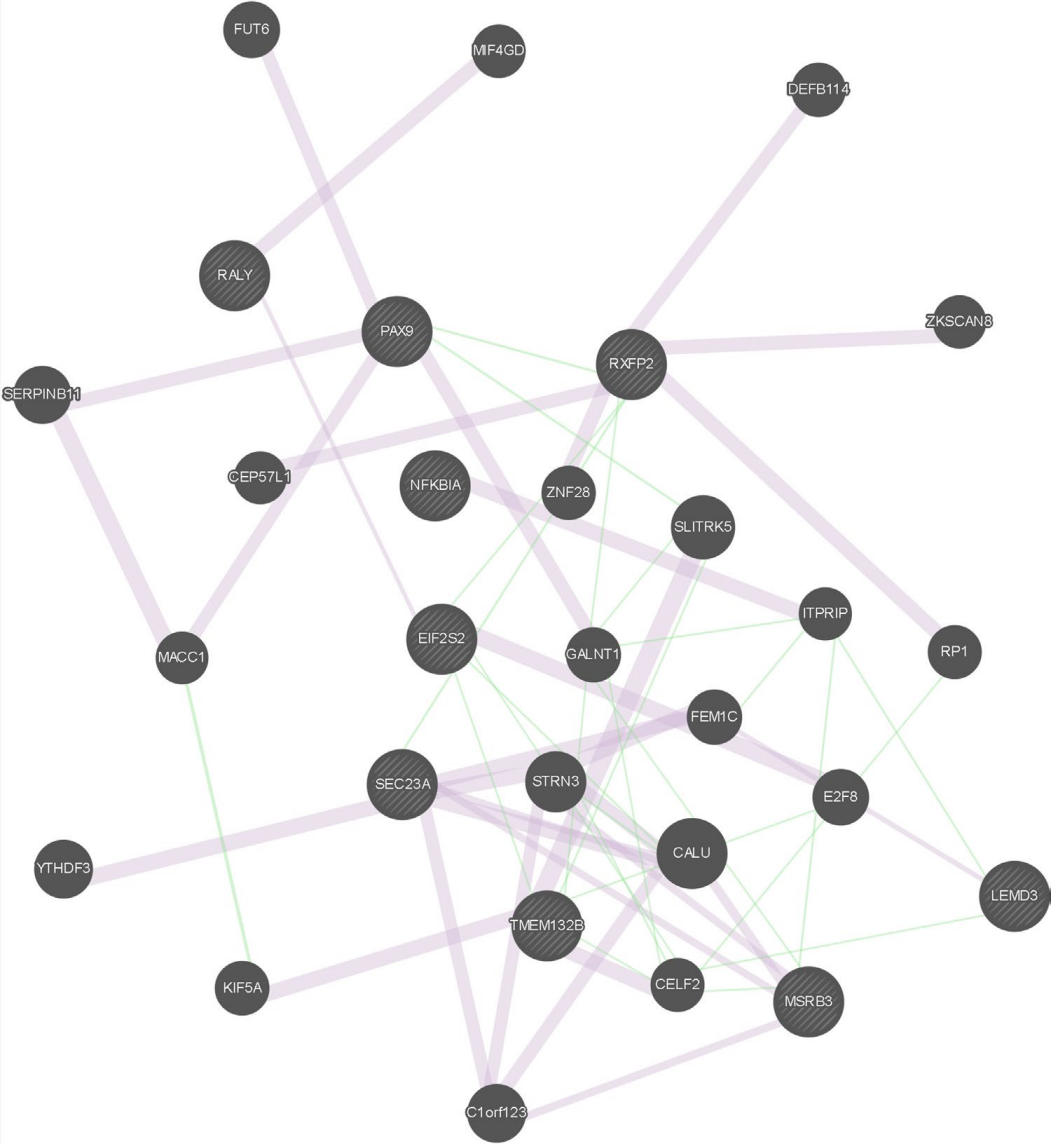
Graphical representation of the gene network analysis. Query genes, i.e., genes detected in this study as putatively selected, are indicated with stripes. Links in light purple indicate the network category co-expression (two genes are linked if their expression levels are similar across conditions in a gene expression study. Most of these data are collected from GEO, the Gene Expression Omnibus); only data associated with a publication are collected. Links in green indicate the network category genetic interaction. (Two genes are functionally associated if the effects of perturbing one gene were found to be modified by perturbations to a second gene. These data are collected from primary studies and BioGRID).

The results of the gene network analysis for the genes located in the putatively selected regions mentioned above are presented in **Figure 1** and **Table S13**. A total of 57 links are reported for the considered 29 genes, out of which 9 genes had been detected in this study as putatively selected. Interestingly, links were observed not only between genes detected as putatively selected using the same approach, either LAMP or the F_ST_-outlier, but also between genes detected as putatively selected using different approaches (SEC23A/MSRB3, RXFP2/PAX9, EIF2S2/TMEM132B, SEC23A/RXFP2), thus suggesting their complementarity in selection signature detection.

### Runs of Homozygosity

Several genomic regions that frequently appeared in a ROH were identified within each breed. **Table 3** provides the chromosome position, and the start and end of the detected ROH islands. The top 0.999 SNPs of the percentile distribution of locus homozygosity values led to the use of different thresholds for each breed (from 0.166, in Gentile di Puglia, to 0.261, in Chinese Merino). The genomic distribution of ROH islands was clearly non-uniform among breeds. Gentile di Puglia showed the highest number (21) of ROH islands, followed by the Spanish Merino (12). Gentile di Puglia, together with Sopravissana, also displayed large proportions (33.3% and 50%, respectively) of ROH islands longer than 5 Mb. These results may well reflect the serious bottlenecks experienced by these breeds in the last 70 years. Three overlapping ROH islands were observed between breed pairs. Spanish Merinos and Gentile di Puglia breeds showed a common 5 Mb genomic region on OAR12 (47,013,871 to 52,019,776 bp). Smaller (<1 Mb) genomic regions were shared between Gentile di Puglia and Chinese Merino on OAR2 (99,442,430 to 100,215,565 bp) and between Chinese Merino and Appenninica on OAR16 (30,100,068 to 30,670,323 bp).

**Table 3:** List of genomic regions of extended homozygosity (ROH islands) identified in the considered Merino and non-Merino breeds.

Breed (locus homozygosity threshold)	OAR	Start	End	N.
Australian Merino (0.196)	3	33,232,651	34,050,238	19
	25	19,861,459	20,568,885	15
	25	21,904,797	22,347,925	13

Spanish Merino (0.231)	1	249,023,519	249,191,465	5
	6	32,912,993	35,003,625	47
	6	37,126,564	38,480,285	30
	6	39,589,194	39,715,842	4
	6	40,342,592	43,655,868	72
	7	1,830,665	3,913,607	45
	12	31,598,245	34,784,182	72
	12	38,121,281	38,765,181	15
	12	41,659,697	42,066,590	8
	12	47,013,871	52,019,776	93
	12	53,371,161	56,327,304	61
	12	63,599,219	64,794,499	26

Gentile di Puglia (0.166)	1	211,018,133	216,875,491	107
	1	270,012,629	271,410,339	28
	2	99,442,340	101,718,337	110
	2	202,780,179	203,472,364	20
	2	217,829,936	223,681,332	116
	2	223,981,060	226,600,106	55
	2	240,008,834	243,300,137	61
	3	211,410,359	218,603,858	139
	5	93,955	3,046,488	66
	5	25,728,102	28,636,862	65
	10	13,800,857	13,988,776	5
	10	14,165,558	20,000,839	111
	10	20,273,388	23,396,844	64
	12	44,162,620	52,019,776	154
	12	70,838,617	78,861,071	151
	17	8,532,536	9,566,661	21
	17	17,289,600	17,844,323	10
	18	3,363,915	6,142,721	47
	22	11,697,681	12,751,792	20
	26	8,124,065	13,498,474	91
	26	17,062,097	19,422,382	38

Sopravissana (0.208)	5	65,184,537	69,108,780	79
	5	72,592,808	73,484,347	20
	5	73,814,249	81,172,608	150
	15	17,158,900	22,517,143	94
	22	18,932,514	24,872,911	109
	22	28,773,373	30,395,735	29

Chinese Merino (0.261)	2	92,669,379	95,401,516	60
	2	95,689,756	100,215,565	82
	3	142,710,943	142,862,611	3
	6	30,411,203	30,508,550	5
	10	67,762,612	70,157,217	45
	16	30,100,068	30,670,323	16

Churra (0.229)	8	32,122,858	34,554,414	49

Ojalada (0.167)	21	17,001,944	19,824,196	44

Bergamasca (0.167)	2	10,823,174	12,893,239	45
	9	36,932,939	37,952,215	24
Appenninica (0.208)	4	44,524,519		1
	16	26,703,405	30,695,539	88

Tibetan (0.243)	8	26,853,601	30,314,835	77
	23	77,105,889	83,429,082	103
	23	90,336,146	94,196,356	64
	23	98,904,521	101,871,791	52
	23	104,351,418	108,119,862	57
	23	114,615,110	120,163,589	90
	23	121,125,567	125,963,555	81

OAR, sheep chromosome. N, number of SNPs.

When comparing the ROH islands observed within each Merino (or Merino-derived) breed (**Table 3**) with regions detected by “local ancestry” analysis involving the same Merino (or Merino-derived) breed (**Tables S4**–**S7**), we found little overlapping. In particular, the two ROH islands detected on OAR25 in Australian Merino were both included in the LAMP region detected for the same breed on the same chromosome. Similarly, the four ROH islands detected on OAR6 in Spanish Merino were all included in the LAMP region detected for the same breed on the same chromosome. No overlapping was observed for Chinese Merino and Sopravissana.

When comparing the ROH islands observed within each Merino (or Merino-derived) breed (**Table 3**) with SNPs detected as significantly differentiated in “F_ST_-outlier” pair-wise contrasts involving the same Merino (or Merino-derived) breed (**Table S8**), some overlapping was observed. In particular, the SNP rs408794746 (34,050,238 bp in OAR3) was significantly differentiated when contrasting Australian Merino with Churra and Ojalada and was also detected within a ROH island in Australian Merino. The significantly differentiated SNP rs425817109 (34,390,603 bp) in the Spanish Merino *vs*. Churra comparison, and the SNP rs400309388 (34,699,452 bp) in the Spanish Merino *vs*. Ojalada comparison, were both included within a ROH island detected in Spanish Merino (OAR12). The SNP rs403786137 (215,181,085 bp in OAR1) in the Gentile di Puglia *vs*. Bergamasca comparison was included within a ROH island detected in Gentile di Puglia. The SNP rs398231484 (216,225,845 bp in OAR3) in the Gentile di Puglia *vs*. Appenninica comparison was included within a ROH island detected in Gentile di Puglia. The SNP rs407100968 (45,671,005 bp) in the Gentile di Puglia *vs*. Appenninica comparison and the SNP rs417849493 (48,709,065 bp) in the Gentile di Puglia *vs*. Bergamasca comparison were both included within a ROH island detected in Gentile di Puglia (OAR12). The SNP rs399908187 (68,477,988 bp in OAR5) in the Sopravissana *vs*. Bergamasca comparison was included within a ROH island detected in Sopravissana. No significantly differentiated SNP overlapping with ROH islands was detected for the Chinese Merino breed.

## Discussion

### Comparison Among Approaches for Selection Signatures Detection

In this study, we investigated selection signatures in various Merino (and Merino-derived) sheep breeds using two different approaches. While the “F_ST_-outlier” is considered a classical method for identification of regions putatively under differential selection in pairs of breeds (or group of breeds), the analysis of local ancestry, i.e., the genetic ancestry of an individual at a particular chromosomal location, in admixed populations to detect genomic regions where a significant excess of ancestry from a given parental breed exists (also known as “admixture mapping”) is so far a less popular approach. Among the “admixture mapping” approaches, LAMP has some interesting features that prompted us to opt for this method. Unlike algorithms that are based on reference haplotype frequencies for each of the parental populations, for which larger sample sizes are required to capture haplotypic diversity, LAMP relies on reference allele frequencies (Shriner, 2013) and is consequently less affected by a reduced sample size. Also, it operates on sliding windows of contiguous SNPs, using a “majority vote” for each locus, over all windows that overlap with the SNP, in order to decide the most likely ancestral population at the marker. This simple approach has been shown to provide fast and robust estimates (Sankararaman et al., 2008). Despite LAMP has been developed for estimation of the locus-specific ancestry in recently admixed populations, it has been shown to be robust to inaccuracies in the parameter “number of generations since the admixture.” A critical issue, limiting the widespread use of LAMP, is represented by the choice of the external reference samples to be used as proxies for the true ancestral populations, as the latter are generally not available for sampling (Shriner, 2013). In this study, a set of four hypotheses, each including a test breed and two proxies for the parental populations, were formulated based on historical knowledge on the origin of breeds and the inferred proportions of global admixture. These have to be interpreted with caution given the possible influence of complex patterns of historical admixture known among Merino and Merino-derived sheep populations (Ciani et al., 2015). The four hypotheses were hence tested using the algorithm implemented in LAMP. On the other side, for the “F_ST_-outlier” approach, we were able to define, a set of nine pair-wise comparisons by contrasting (i) Merino populations of Iberian origin (Spanish Merino and Australian Merino, respectively) with non-Merino populations of Iberian origin (Churra and Ojalada, respectively), (ii) Merino populations of Italian origin (Gentile di Puglia and Sopravissana, respectively) with non-Merino populations of Italian origin (Appenninica and Bergamasca, respectively), and (iii) a Merino population of Asian origin (Chinese Merino) with a non-Merino population of Asian origin (Tibetan). The rationale behind the above pairing is that differentially selected loci may be easier to detect when contrasting more homogeneous breeds, such as Merino *versus* non-Merino breeds from the closest geographical area (Manunza et al., 2016).

Consistently with expectations, the two adopted approaches produced only partly overlapping signals. Indeed, the two methods rely on different algorithms and different assumptions, which also imposed a different organization of the dataset used with the two approaches (four single-breed tests *vs.* nine pair-wise comparisons, for the “local ancestry” and the FST-outlier” approaches, respectively), thus hampering direct head-to-head comparisons. Notwithstanding, the majority of the regions detected using the “local ancestry” method showed overlapping signals with the “F_ST_-outlier” results. Although we interpreted the above as evidence supporting the robustness of the obtained results, it must be taken into consideration that regions identified by “local ancestry” were generally large, and significant SNPs detected *via* the “F_ST_-outlier” method may likely occur in there by chance. Indeed, the number of loci identified as putatively under selection pressure using the “F_ST_-outlier” method largely exceeds the number of putatively selected regions identified using the “local ancestry” approach.

In this study, we also investigated genomic regions of high homozygosity (ROH islands), as these have been shown to be abundant in regions under positive selection (Metzger et al., 2015; Mastrangelo et al., 2017; Purfield et al., 2017; Talenti et al., 2017a; Talenti et al., 2017b; Mastrangelo et al., 2018). While we observed little overlapping between ROH islands and regions identified *via* “local ancestry,” some overlapping was observed between ROH islands and SNPs detected as significant using the “F_ST_-outlier” approach. ROH islands may be the consequence of the genetic hitchhiking phenomenon at loci physically linked to the variant site under direct positive selection pressure. The “local ancestry” approach looks for regions with an excess of ancestry from one of the two parental populations, and not necessarily these regions have to display high homozygosity, although this feature is likely to be observed in case of a strong selective sweep. Similarly, in the “F_ST_-outlier” approach, homozygous genotypes (for different alleles in the two breeds) at loci physically linked to the variant site under direct positive selection pressure may display high frequencies if a strong differential selection existed among the two considered breeds. Also, the argumentation reported above may apply here: the larger number of loci identified as putatively under selection pressure using the “F_ST_-outlier” method may be more likely to occur by chance within large ROH islands compared to the fewer genomic regions identified *via* “local ancestry.” Moreover, ROH analysis might detect selection related to any trait, while contrasting Merino and non-Merino is more likely to detect signals related to this specific trait. Finally, the existence of ROH islands may be due to non-genetic factors such as demography.

### Best Candidate Regions and Putatively Selected Genes

As the aim of this study was to identify loci most likely associated with the Merino phenotype, we arbitrarily identified (i) the best candidate regions detected *via* the “local ancestry” approach as those being shared by all the four breeds and (ii) the best candidate SNPs detected *via* the “F_ST_-outlier” approach as those observed in at least 70% of the pair-wise comparisons (six out of nine). Based on the above, two large regions on OAR17 and OAR18 were retained for (i), and three, on OAR3, OAR10, and OAR13, for (ii). The robustness of the adopted procedure was also suggested by the occurrence, in all of the five regions, of QTLs/associations known to be related to wool traits in the ovine species. Moreover, at OAR17, combining analysis of “local ancestry” and inspection of the sheep QTL database allowed to significantly shorten the candidate interval. On the contrary, known QTLs for wool traits described on OAR18 and OAR3 are mapped within extremely large chromosome intervals. These were identified by Allain et al. (2006) and Ponz et al. (2001) who performed whole-genome scans using microsatellite markers on experimental flocks obtained crossing Lacaune with Sarda, and Berrichon du Cher (a Merino-derived breed) with Romanov (a non-Merino breed), respectively. In what follows, the gene content of the best candidate regions is presented by chromosome order.

#### OAR3

The region detected on OAR3 contains five genes, *LOC105609945* (long noncoding RNA), *MSRB3* (methionine sulfoxide reductase B3), *LOC105609947* (long noncoding RNA), *LOC105609948* (a pseudo-gene), and *LEMD3* (LEM domain containing 3). Interestingly, the sub-region containing the genes *MSRB3*, *LOC105609947*, and *LEMD3* was found to harbor a selection signature putatively for tail fat deposition in previous studies contrasting thin- *vs.* fat-tail sheep breeds, from China (Yuan et al., 2017), and from North Africa and the Chios island (Mastrangelo et al., 2019), for adaptation when contrasting the Red Maasai sheep with the Ethiopian Menz (Fariello et al., 2014), and for ear morphology in Chinese sheep breeds (Wei et al., 2015) and in French Suffolk sheep (Rochus et al., 2018). The latter suggest to consider the genes encoded by the signal on OAR3, notably MSRB3 and LEMD3, as candidates for ear size based on literature showing the possible role of the two genes in ear position in dogs (Vaysse et al., 2011) and ear size in pigs (Wilkinson et al., 2013). A more detailed discussion of each single gene in the OAR3 selection signature is provided below.

*LOC105609945*—No evidence for involvement of *LOC105609945* in any peculiar Merino feature was found.

*MSRB3*—The methionine sulfoxide reductase B3 (*MSRB3*, alias *DFNB74*) catalyzes the reduction of free and protein-bound methionine sulfoxide to methionine. This antioxidant repair enzyme has been described in human epidermal keratinocytes and melanocytes, as well as in hair follicles (Taungjaruwinai et al., 2009). It has been shown to be expressed also in inner and outer hair cells of mouse inner ear (Ahmed et al., 2011). Diseases associated with *MSRB3* include deafness (https://www.genecards.org). Down-regulation of *MSRB3* has been shown to impair the normal auditory system development through hair cell apoptosis in zebrafish (Shen et al., 2015). The gene has been found in previous selection signatures studies in sheep (Kijas et al., 2012; Fariello et al., 2014; Wei et al., 2015; Manunza et al., 2016; Yuan et al., 2017; Rochus et al., 2018; Mastrangelo et al., 2019). More interestingly, for this study, it has been found within a selection signature observed contrasting fine-wool Merino and coarse-wool Churra sheep breeds (Gutiérrez-Gil et al., 2017). Another line of evidence for the involvement of *MSRB3* in hair/wool physiology comes from the observation that actin’s polymerization properties and actin cytoskeletal-mediated events, such as correct bristle development, which are altered by specific oxidation of its conserved methionine (Met)-44 residue on the pointed-end of actin subunits, are rescued by a methionine sulfoxide enzyme reductase (SelR/MsrB) in *Drosophila* (Hung et al., 2013). In this species, actin plays a role not only in bristle but also in wing hair development (Guild et al., 2005). In mammals, actin has been shown to be one of the major components of both the water-soluble and -insoluble fraction from hair and hair follicles (Vermorken et al., 1981; Lee et al., 2006). Actin bundles in the hair follicle would act as stress fibers and serve as a tensile scaffold for the growth and integrity of the hair follicle (Furumura and Ishikawa, 1996). In Tibetan sheep, microRNAs differentially expressed in wool follicles during anagen, catagen, and telogen phases, thus potentially regulating wool follicle development, targeted, among others, genes in the pathways that regulate the actin cytoskeleton (Liu et al., 2013). *MSRB3* also contained the (intronic) SNP that, in this study, displayed the highest number of “F_ST_-outlier” pair-wise comparisons showing signal of selection observed in the OAR3 region.

*LOC105609947*—No evidence for involvement of *LOC105609945* in any peculiar Merino feature was found.

*LOC105609948*—It’s a pseudo for the ubiquitin-conjugating enzyme E2 D3 gene (*UBE2D3*), which is part of the bone morphogenic protein (BMP) signaling pathways (gene ontology database accession ID: GO:0030509). BMP ligands (*BMP2* and *BMP4*) when expressed in dermal macro-environment during telogen (resting phase of hair cycle) have been shown to strongly suppress ability of resting hair follicles to be reactivated and grow again (International Patent no. WO2010059861A1 available at https://patents.google.com/patent/WO2010059861A1).

*LEMD3*—As previously mentioned, the LEM domain containing three gene (alias *MAN1*) has been found in various selection signatures studies in sheep (Fariello et al., 2014; Wei et al., 2015; Manunza et al., 2016; Yuan et al., 2017; Rochus et al., 2018; Mastrangelo et al., 2019), including the study by Gutierrez-Gil et al. (2017) where fine-wool Merino and coarse-wool Churra sheep breeds were contrasted. Moreover, it has been found associated with the abnormal hair quantity phenotype from the HPO Gene-Disease Associations dataset (Köhler et al., 2014).

#### OAR10

The region detected on OAR10 includes four genes: *LOC106991357* (long noncoding RNA), *LOC101110773* (elongation factor 1-alpha 1-like), *RXFP2* (relaxin/insulin-like family peptide receptor 2), and *LOC106991379* (a pseudo-gene). Despite this region was detected as putatively selected in studies investigating tail fat deposition (Moioli et al., 2015; Yuan et al., 2017; Mastrangelo et al., 2019) and adaptation (Yang et al., 2016; Seroussi et al., 2017), RXFP2 is the most studied gene and is well known for being involved in horn presence/absence and morphology in sheep (Johnston et al., 2011; Kijas et al., 2012; Fariello et al., 2014; Pan et al., 2018). A genome-wide association study for wool traits in Chinese Merino sheep detected, on this chromosome, a significant SNP for “fiber diameter” at position 30 Mb, together with several SNPs significant for “crimp” at 26–27 Mb (Wang et al., 2014). In what follows, a more detailed discussion of the four genes annotated in the OAR10 region is provided.

*LOC106991357*—No evidence for involvement of this locus in any peculiar Merino feature was found.

*LOC101110773*—It codes for an elongation factor 1-alpha 1-like. The elongation factor 1-alpha 1 (*EF1A1*) is a GTP-binding protein which has a primary function as an essential house-keeping gene by delivering aminoacyl-tRNAs to the ribosome during the elongation step of protein translation. *EF1A1*, together with genes associated to the Usher syndrome, a congenital disease characterized by perturbation of normal organization and growth of hair bundles within the inner ear, is a downstream target of *GBX2*, which induces *EF1A1* activation upon binding to the *EF1A1* core promoter. *GBX2* has been shown to be expressed in the otic placode, which develops into the inner ear. Loss-of-function and mis-expression studies have shown that *GBX2* is essential for development of the inner ear sensory organs. However, neither direct evidence for involvement of *EF1A1* in hair bundles organization and growth nor in any peculiar Merino phenotype has been found so far. Another elongation factor type (*EF1Bγ*) has been proposed to bind to keratin (Kim et al., 2006). The presence of large amounts of *EF1Bγ* in keratin bundle rich hair fibers would support its biological role in the intermediate filament organization (Sasikumar et al., 2012).

*RXFP2*—This gene is involved, among others, in the biological process “activation of adenylate cyclase activity” (https://www.uniprot.org/uniprot/Q8WXD0). Adenylate cyclase is responsible for the synthesis of 3’,5’-cyclic adenosine monophosphate (cAMP). Agents that increase cAMP levels have been shown to be potent inhibitors of human and mouse hair follicle growth (Harmon and Nevins, 1997). However, we cannot exclude that, in our tested scenarios, different alleles at this gene may have been differentially selected in the considered breeds as a consequence of selection toward different horn phenotypes. Rochus et al. (2018) highlighted that a number of single nucleotide polymorphisms exist in French sheep in the region extending 100 Kb upstream of *RXFP2*, with haplotypes in polled sheep being distinct from those observed in horned sheep. From these findings, they suggested that multiple ancient mutations, rather than a single mutation, are likely affecting horn phenotypes. Pan et al. (2018) reported strong association between three SNPs within the *RXFP2* gene and horn sizes in a Tibetan population characterized by the presence of animals with heterogeneous horn types.

*LOC106991379*—No evidence for involvement of this locus in any peculiar Merino feature was found.

It is worth mentioning that, only 0.076 Mb upstream to LOC106991357 on OAR10, the gene *FRY* is mapped (interval 28,959,450–29,212,913 bp). Looking at our results separately for each tested scenario, we observed that this interval was overlapping with the region detected by the “local ancestry” method in Chinese Merino, as well as with the regions detected by the “F_ST_-outlier” method in the Chinese Merino *vs.* Tibetan, Australian Merino *vs.* Churra, and Gentile di Puglia *vs*. Appenninica comparisons. Moreover, the *FRY* interval was only slightly upstream to the regions detected by the “F_ST_-outlier” method in the Australian Merino *vs.* Ojalada (0.2 Mb), Spanish Merino *vs.* Ojalada (0.18 Mb), Gentile di Puglia *vs.* Bergamasca (0.18 Mb), Sopravissana *vs.* Appenninica (0.2 Mb), and Sopravissana *vs*. Bergamasca (0.18 Mb). In sheep, *FRY* has been suggested as a key candidate gene for the piebald phenotype in Merino (Garcia-Gamez et al., 2011) and has been suggested to be associated with the black spot phenotype in Valley-type Tibetan sheep (Wei et al., 2015), and with differences in coat color pigmentation distribution between the Awassi and Afec-Assaf sheep (Seroussi et al., 2017). On the contrary, Zhang et al. (2013) detected *FRY* when contrasting Rambouillet and Suffolk sheep and suggested it to be a candidate gene affecting wool quality. Indeed, *FRY* encodes a protein furry homolog that, in *Drosophila*, has been found in growing hairs (He et al., 2005), and whose disruption has been shown to provoke abnormally branched bristles and strong multiple-hair phenotype, with clusters of epidermal hairs and branched hairs (Cong et al., 2001). Fang et al. (2010), following the transgenic *FRY* protein *in vivo*, found it to be highly mobile and to accumulate at the distal tip of growing bristles and suggest that it could function in directing/mediating the intracellular transport needed for polarized growth.

#### OAR13

The region detected on OAR13 contains a single gene (*RALY*). It encodes a heterogeneous nuclear ribonucleoprotein that binds poly-U-rich elements within several RNAs and regulates the expression of specific transcripts (Cornella et al., 2017; Rossi et al., 2017). In early 90s, the gene was shown to be involved in the pleiotropic lethal yellow phenotype of the mouse due to a deletion of the genes *RALY* and *EIF2S2* (eukaryotic translation initiation factor 2 subunit 2), upstream the *ASIP* (agouti signaling protein) gene, responsible for the ectopic over-expression of the agouti signaling protein under the control of the *RALY* promoter (Michaud et al., 1993; Duhl et al., 1994). The gene has been repeatedly detected, often together with the neighbor ASIP gene, in association studies concerning skin pigmentation and skin neoplasms, (https://www.ncbi.nlm.nih.gov/gap/phegeni?tab=1&amp;gene=22913; Jacobs et al., 2015), as well as in several other type of cancers, where it is considered to represent a metastatic marker (Roberts et al., 2019). In 2013, a mutation in this gene has been associated with the saddle tan and black-and-tan phenotypes in Basset Hounds and Pembroke Welsh Corgis (Dreger et al., 2013). Similarly, it has been associated with coat color phenotypes in Chinese and Iranian goats (Guo et al., 2018; Nazari- Ghadikolaei et al., 2018). Worth mentioning that a SNP at OAR13 (position 62.9 Mb) was associated by Bolormaa et al. (2017) with wool fiber diameter in Merino sheep. Although the SNP is not far from the *RALY* gene, it mapped within the *EIF2S2* gene, which has been shown to be involved in protection against chemotherapy-induced alopecia (Nasr et al., 2013).

#### OAR17

Out of the two pairs of genes flanking the two QTLs on OAR17, one (*LOC101120019*) is a pseudo-gene related to a 60S ribosomal protein (L10A), one (*TMEM132B*) codes for a trans-membrane protein, one (*LOC101115905*) codes for refilin-A (alias *FAM101A*), and one (*LOC106991703*) is responsible for the production of a long noncoding RNA. Their possible involvement in the Merino phenotype is discussed here based on evidences from the literature.

*LOC101120019*—A mutation in a 60S ribosomal protein (L21) has been shown to be involved in hereditary hypotrichosis simplex (HHS), a form of nonsyndromic inherited hair loss disorders (Zhou et al., 2011). 60S ribosomal proteins (L6 and L24) have been shown to be expressed in human anagen hair samples (Carlson et al., 2018). Interestingly, the 60S ribosomal protein L10A has been shown to be expressed in root hairs of *Medicago truncatula* (Covitz et al., 1998). As is common for genes encoding ribosomal proteins, multiple processed pseudo-genes of the 60S ribosomal protein L10A are dispersed through the genome (https://www.ncbi.nlm.nih.gov/gene/4736). In particular, *LOC101120019* on OAR17, being a pseudo-gene, is more likely to play, if any, a regulatory function on the hair physiology.

*TMEM132B*—The trans-membrane protein 132B is required for normal inner ear hair cell function (https://www.genecards.org/cgi-bin/carddisp.pl?gene=TMEM132E). *TMEM132A*, but not *TMEM132B*, *TMEM132C*, or *TMEM132D*, was found to be expressed in wool follicle bulb of Tibetan sheep during phase transformation from the middle anagen, to catagen and late telogen/early anagen (Liu et al., 2015). *TMEM132E* was found to be highly expressed in murine inner hair cells, and a variant in *TMEM132E* was identified as the most likely cause of autosomal-recessive nonsyndromic hearing loss. Knockdown of the *TMEM132E* ortholog in zebrafish affected the mechanotransduction of hair cells. (Li et al., 2015).

*FAM101A*—The gene product is involved in the regulation of the perinuclear actin network and nuclear shape through interaction with filamins. It plays an essential role in the formation of cartilaginous skeletal elements (UniProtKB:Q5SVD0). In addition, it has been shown to be differentially expressed in hair follicle stem cells residing in the bulge of mouse hair follicles *versus* the epithelial basal cells outside the bulge (Chang, 2014). *FAM101A* mRNA was detected *via* next-generation sequencing in wool follicle bulb samples of Tibetan sheep from middle anagen, catagen, and late telogen/early anagen phases (Liu et al., 2015). In a genome-wide association study performed using 50 K SNPs in a Baluchi sheep population, one of the significant SNP markers associated with greasy fleece weight was located within *FAM101A* (Ebrahimi et al., 2017).

*LOC106991703*—No evidence for involvement of *LOC106991703* in any peculiar Merino feature was found.

#### OAR18

The region identified on OAR18 included a large number of genes (70) that obviously hampered a detailed analysis of the available literature for each single gene. We hence opted for checking which of the above genes could be retrieved by querying the human GeneCards database using keywords representative of Merino phenotypes. While none of the genes was retrieved when using “wool” or “horn” keywords, 16 genes were retrieved when using the keyword “hair” and, among them, three displayed particularly high GeneCards relevance scores (*NFKBIA, SEC23A*, and *PAX9*).

*NFKBIA* (NFKB inhibitor alpha)—It has been shown to modulate *WNT*, *SHH*, and *LHX2IS* signaling at early stages of hair follicle development in mice. In particular, in the epidermis of mice lacking the transcription factor nuclear factor-kappa B activity, primary hair follicle pre-placode formation is initiated without progression to proper placodes (Tomann et al., 2016). The gene has been also detected as putatively under selection in a Chinese Merino sheep population (Liu et al., 2017).

*SEC23A*—It is one of the major components and markers of COPII vesicles from endoplasmic reticulum. It has been found associated with the sparse hair phenotype in humans (https://mseqdr.org/hpo_browser.php?8070) Moreover, it may contain causative mutations for an autosomal recessive disease known as cranio–lenticulo–sutural dysplasia, *alias* Boyadjiev–Jabs syndrome, in which patients have abnormal hair, among other cranio-facial abnormalities. Also, it has been shown to co-localize with the three proteins, transmembrane (Cdh23), scaffold (harmonin), and actin-based motor (Myo7a), whose defect is responsible of various types of the Usher syndrome, a multi-genic congenital disease characterized by perturbation of normal organization and growth of hair bundles within the inner ear (Blanco-Sánchez et al., 2014)

*PAX9*—It is a member of the paired box (PAX) family of transcription factors. Heterozygous mutations in *PAX9* have been associated in humans with non-syndromic tooth agenesis, non-syndromic, and familial oligodontia, with peg-shaped laterals and microdontia incisors. Often, these symptoms are associated with hair defects (Roberts and Chetty, 2018) as the same genes responsible for tooth development are involved in the growth and development of the other tissues derived from the ectoderm, including hair.

In general, biological evidence for the involvement in plausible Merino phenotypes was observed for the vast majority of coding genes in putatively selected regions detected either *via* “local” ancestry” or the F_ST_-outlier” approach. The above result highlights the power of the multi-cohort approach adopted here. While we cannot exclude that false positive signals may have been retained in this study, still this represents so far the most complete genome-wide study of selection signatures for the Merino phenotype. The selection signatures reported here provide a comprehensive insight into the genetic basis underlining the Merino phenotype in sheep, which appeared here to be mainly represented by wool (and horn) traits. Targeted studies at both physiological and molecular levels will be needed to better understand the biological complexity behind these commercially relevant traits.

## Data Availability Statement

The whole SNP genotype dataset is available on the WIDDE database (http://widde.toulouse.inra.fr/widde/).

## Author Contributions

Conception of the work: SMe, EC. Data analysis: SMe, SMa, EC. Results interpretation: SMe, SMa, JL, EC. Drafting the article: SMe, EC. Critical revision of the article: SMe, SMa, MH, JL, EC. Final approval of the version to be published: SMe, SMa, MH, JL, EC.

## Conflict of Interest

The authors declare that the research was conducted in the absence of any commercial or financial relationships that could be construed as a potential conflict of interest.

The handling editor declared a past co-authorship with one of the authors SMa.

## Supplementary Material

Data Sheet 1 Information on the considered breeds.

Figure S1 Individual proportions of global admixture for the four considered datasets. Dataset composition: (A) Rambouillet, Chinese Merino, Tibetan; (B) Australian Merino, Sopravissana, Appenninica; (C) Gentile di Puglia, Spanish Merino, Appenninica; (D) Gentile di Puglia, Australian Merino, Appenninica. To estimate admixture proportions of the four test breeds (A, Chinese Merino; B, Sopravissana; C, Spanish Merino; D, Australian Merino), each dataset was assumed to be arranged into two sub-populations. Color codes define the admixture proportions for each animal. Individual proportions of global admixture were averaged within breed to obtain a, the fraction of global admixture, adopted as parameter in the “local ancestry” analyses (see main text).

Table S1 Sheep breeds considered throughout this study. N, number of genotyped animals.

Table S2 Sheep breeds considered in the four different scenarios tested using the “local ancestry” approach. N, number of genotyped animals; α, fraction of global admixture.

Table S3 Sheep breeds considered in the nine pair-wise comparisons tested using the “FST-outlier” approach.

Table S4 Putatively selected regions identified in Australian Merino using the “local ancestry” approach. Merino genome-wide marker average ancestry (MAA) for this tested scenario was 0.87. For each region, the sheep chromosome (OAR), the name and the position, expressed in base pairs (bp), of the start and end SNPs (SNP ID), together with the MAA values representing the Merino fraction for the start and end SNPs, are provided. In bold and italics the region overlapping with ROHs detected within the Australian Merino breed. Highlighted in light yellow, the regions where at least one significant SNP in at least one pair-wise comparison of the “FST-outlier” method involving the corresponding Merino (or Merino-derived) breed was observed (see main text).

Table S5 Putatively selected regions identified in Chinese Merino using the “local ancestry” approach. Merino genome-wide marker average ancestry (MAA) for this tested scenario was 0.91. For each region, the sheep chromosome (OAR), the name and the position, expressed in base pairs (bp), of the start and end SNPs (SNP ID), together with the MAA values representing the Merino fraction for the start and end SNPs, are provided. Highlighted in light yellow, the regions where at least one significant SNP in at least one pair-wise comparison of the “FST-outlier” method involving the corresponding Merino (or Merino-derived) breed was observed (see main text).

Table S6 Putatively selected regions identified in Sopravissana using the “local ancestry” approach. Merino genome-wide marker average ancestry (MAA) for this tested scenario was 0.76. For each region, the sheep chromosome (OAR), the name and the position, expressed in base pairs (bp), of the start and end SNPs (SNP ID), together with the MAA values representing the Merino fraction for the start and end SNPs, are provided. Highlighted in light yellow, the regions where at least one significant SNP in at least one pair-wise comparison of the “FST-outlier” method involving the corresponding Merino (or Merino-derived) breed was observed (see main text).

Table S7 Putatively selected regions identified in Spanish Merino using the “local ancestry” approach. Merino genome-wide marker average ancestry (MAA) for this tested scenario was 0.77. For each region, the sheep chromosome (OAR), the name and the position, expressed in base pairs (bp), of the start and end SNPs (SNP ID), together with the MAA values representing the Merino fraction for the start and end SNPs, are provided. In bold and italics the region overlapping with ROHs detected within the Spanish Merino breed. Highlighted in light yellow, the regions where at least one significant SNP in at least one pair-wise comparison of the “FST-outlier” method involving the corresponding Merino (or Merino-derived) breed was observed (see main text).

Table S8 Results of the analyses performed using the “FST-outlier” approach for the nine pair-wise comparisons between Merino and non-Merino breeds. The sheep chromosome (OAR), the name (SNP ID) and the position, expressed in base pairs (bp), of the significant loci displaying qval < 0.05, and the corresponding FST values, are shown. In bold and italics, loci included within ROH islands detected in the same Merino (or Merino-derived) breed (see main text). Highlighted in light yellow, loci located within putatively selected regions identified, for the corresponding four considered breeds (Australian Merino, Spanish Merino, Sopravissana, Chinese Merino) using the “local ancestry” approach.

Table S9 Gene content of the region on OAR17 detected as putatively selected by “local ancestry” analysis. N., sequential numbers. Gene ID, gene symbol.

Table S10 Gene content of the region on OAR18 detected as putatively selected by “local ances

Table S11 Quantitative Trait Loci (QTLs) known in sheep and mapped to regions detected in this study as putatively selected via “local ancestry” (A) and “FST-outlier” (B) analysis. QTL ID, QTL accession code at the NCBI Sheep Genome Data Viewer. OAR, sheep chromosome.

Table S12 Gene content of the regions on OAR3 (A), OAR10 (B), OAR13 (C) and OAR19 (D) detected as putatively selected by “FST-outlier” analysis. N., sequential number. Gene ID, gene symbol.

Table S13 Results of the gene network analysis. In italics, interactions between genes detected as putatively selected using the same approach (either LAMP or FST-outlier). In bold, interactions between genes detected as putatively selected using different approaches.
