# Supplementary material for: A Combined Multi-Cohort Approach Reveals Novel and Known Genome-Wide Selection Signatures for Wool Traits in Merino and Merino-Derived Sheep Breeds
Source: Front Genet. 2019 Oct 25;10:1025. doi: 10.3389/fgene.2019.01025 (PMC6824410; doi:10.3389/fgene.2019.01025)
Supplement: Data Sheet 1 — Information on the considered breeds. [file DataSheet_1.docx]

**Data Sheet 1.** Information on the considered breeds.

*Merino and Merino-derived sheep breeds*

Australian Merino - This breed has emerged from the Spanish Merino variety that was landed firstly in Australia in 1797 (National Museum of Australia). It's a medium-sized breed much prized for its primary production of soft, bright, white fleece (fine wool) with high crimp frequency. Australian Merino is one of the main sheep breed of Australia (Australian Bureau of Statistics, 1999). Accounting to fiber diameter parameter, four major classes could be distinguished: Fine wool (19 microns), Fine-medium wool (20-22 microns), superfine wool (18 microns), and strong wool (23-25 microns) (Australian Bureau of Statistics, 1999). Their distribution throughout Australia is determined by the climatic, geographic, and management factors (intensive rate); the finer-woolled types thrive well, broadly, in cooler areas. Rams usually present long spiral horns while ewes are pooled. The breed is also known for its longevity and strong flocking instinct.

Spanish Merino - Classified as a native Spanish breed originated from Iberia (Porter et al., 2016), Spanish Merino has marked exhaustively the history of sheep wool since the beginning of 12^th^ century (FAO, 1978) and is evaluated as the ancestor of all other Merino breeds that were disseminated throughout the world (Ciani et al.2015). In more recent time, Spanish Merino has been exploited as a triple-purpose sheep (primary for wool, then, for meat and milk). Actually, the breed is mostly found in Extremadura, western Andalusia and some parts of Castile (Porter et al., 2016). Its reputation comes from the peculiarity of producing abundant fleece–eventually in white or black variety–strongly crimped (6-12 crimps/cm); the fiber diameter could be ranged from 18 to 25 microns with 6 cm of staple length (Porter et al., 2016). Mature rams usually have spiral ridged horns and could weigh up to 80-85 Kg with a shoulder height of 82 cm, contrary to ewes that are polled, more lighter (50-55 Kg) and shorter in shoulders (70 cm) (Porter et al., 2016).

Gentile di Puglia - The breed is thouth to have originated from an early crossing of indigenous ewes with Spanish Merino rams, occurred in the province of Foggia, in the Puglia region (Southern Italy) (Sarti et al., 2006; Ciani et al., 2015). Rams are horned with spiral ridged horns and ewes are polled. The breed has been reared mainly for its fine wool; rams could produce 6 Kg of wool, with a staple length of 7-9 cm and a fiber diameter of 19-23 microns (with a slight yellow tone) (Porter et al., 2016), and only 3.5 Kg for ewes (Breeds of Livestock, 2019). The fleece showed good homogeneity and the quality of wool have a tendency to be better in young animal (Sarti et al., 2006). The wool covers the white face and legs. Additionally, Gentile di Puglia is even used for its secondary productions (meat and milk).

Sopravissana – Similarly to Gentile di Puglia, Sopravissana breed emerged from a sporadic introgression but from different Merino stocks: the French Merino–Rambouillet rams, crossed with native Vissana ewes of central Italy (region of Visso from the province of Macerata) (Lasagna et al., 2011; Lancioni et al., 2013; Ciani et al., 2015). The breed shared the main features shown in Gentile di Puglia breed, in particular, the developed spiral horns, notably in rams. Notwithstanding the good quality of its wool, the exploitation schema of this breed was converted to meat and milk production, due to the decrease of wool demands in latter decades.

Chinese Merino - Chinese Merino represents the excellent specialized sheep breed for fine and soft wool production in China (Liu et al., 2017). The breed was developed through crossing Soviet Merino and Rambouillet rams with native Chinese ewes, notably Mongolian and Tibetan (Ciani et al., 2015). Considering wool qualities, the average fiber diameter range from 20-25 microns and the average greasy fleece weight, for ewes, reach 5.9 Kg, with a relatively clean yield of 47 to 54 % (FAO, 1985).

Rambouillet - We specify, here, that we talk about the American Rambouillet which could be probably originated from either the sire of French Rambouillet or German Homeyer Flocks (Breeds of Livestock, 2019b). In America (United-States), the breed supports a wide range of temperature which favored to show it kept in various regions, from Texas to Wisconsin (American Rambouillet Sheep Breeders Association, 2019). A mature ram could reach the weight of 135 Kg, while ewe weighs in the range of 68-90 Kg. The fleece weight range is 3.6-8.1 Kg for females with a staple length varying from 5 to 10 cm, and fiber diameter could range from 18.5 to 24.5 microns (Breeds of Livestock, 2019b). The breed is well adapted to produce wool and meat of high quality.

*Non-Merino sheep breeds*

Churra - Churra is a native Spanish sheep breed, well-known for its excellent milk production and mainly raised in Castile and León, two regions from northwestern Spain. The body weight range 65-75 Kg and 45-55 Kg for rams and ewes, respectively (Baro et al., 1995). The breed is rustic, and has white coarse-wool and black-spotted white face.

Ojalada - A native Spanish breed, commonly found in the mountains of Teruel, Guadalajara, Zaragoza, and Soria, as well as, Castellon and Tarragona (Porter et al., 2016). Ewes shared a semiclosed white fleece, a white face showing black pigmentation around the eyes, the ear tips, and muzzle, and could reach 45 Kg of live body weight (Ovigen, 2019). Unusually, rams develop spiral horns. The breed is mostly exploited for its meat production.

Bergamasca - Bergamasca could be found from Lombardy in the Bergamo province to the central part of the Italian Pre-Alps (Riva et al., 2004). The average weight is 110 and 80 Kg for adult rams and ewe, respectively (Riva et al., 2004). The head has a markedly convex profile with long and wide lop ears (Porter et al., 2016). The breed has a white coarse-wool and face and is much appreciated for its meat. In addition, the breed is hornless for both sexes (Riva et al., 2004; Porter et al., 2016).

Appenninica - The breed is mainly raised in central Italy, in Toscana, Emilia, Umbria, and Abruzzi regions (Italian breeds of sheep, 2019). Of a white medium-sized body, rams could reach 77 cm of shoulders height and only 69 cm for ewes (Italian breeds of sheep, 2019). The body weight averaged 78 Kg and 56 Kg for rams and ewes, respectively (Italian breeds of sheep, 2019). Likewise Bergamasca, Appenninca is hornless for both sexes with semi-lopped ears (Breeds of Livestock, 2019a). It's also considered as a coarse-wool meat breed.

Tibetan - A native Chinese sheep breed known to be a carpet-wool breed exploited primarily for its meat. It inhabits the harsh environment of the Qinghai-Tibetan Plateau, living at an altitude of 3.000 to 5.000 m, it is the main resource of meat and milk in those areas, and  the major  source of income for most nomadic and seminomadic people in these regions. The traditional feeding system for Tibetan sheep is free range husbandry (Ren et al., 2019).

**References**

American Rambouillet Sheep Breeders Association (2019). American Rambouillet Sheep Breeders Association. Available at: http://www.countrylovin.com/ARSBA/index.htm [Accessed September 11, 2019].

Australian Bureau of Statistics (1999). *Year Book Australia 1999*. Aust. Bureau of Statistics.

Baro, J., PRIMITIVO, F., VETERINARIA, I., and SPA, L. (1995). Breeding programme for the Spanish Churra sheep breed.

Breeds of Livestock (2019a). Apennine Sheep — Breeds of Livestock, Department of Animal Science. Available at: http://afs.okstate.edu/breeds/sheep/apennine/ [Accessed September 11, 2019].

Breeds of Livestock (2019). Gentile di Puglia Sheep — Breeds of Livestock, Department of Animal Science. Available at: http://afs.okstate.edu/breeds/sheep/gentiledipuglia/ [Accessed September 10, 2019].

Breeds of Livestock (2019b). Rambouillet Sheep — Breeds of Livestock, Department of Animal Science. Available at: http://afs.okstate.edu/breeds/sheep/rambouillet/ [Accessed September 11, 2019].

Ciani, E., Lasagna, E., D’Andrea, M., Alloggio, I., Marroni, F., Ceccobelli, S., et al. (2015). Merino and Merino-derived sheep breeds: a genome-wide intercontinental study. *Genet. Sel. Evol.* 47, 64. doi:10.1186/s12711-015-0139-z.

FAO (1985). *Livestock breeds in China*. Rome: Food and Agriculture Organization of the United Nations.

Italian breeds of sheep (2019). Appenninica. Available at: http://eng.agraria.org/sheep/appenninica.htm [Accessed September 11, 2019].

Lancioni, H., Lorenzo, P. D., Ceccobelli, S., Perego, U. A., Miglio, A., Landi, V., et al. (2013). Phylogenetic Relationships of Three Italian Merino-Derived Sheep Breeds Evaluated through a Complete Mitogenome Analysis. *PLoS ONE* 8. doi:10.1371/journal.pone.0073712.

Lasagna, E., Bianchi, M., Ceccobelli, S., Landi, V., Martínez, A. M., Pla, J. L. V., et al. (2011). Genetic relationships and population structure in three Italian Merino-derived sheep breeds. *Small Rumin. Res.* 96, 111–119. doi:10.1016/j.smallrumres.2010.11.014.

Liu, S., He, S., Chen, L., Li, W., Di, J., and Liu, M. (2017). Estimates of linkage disequilibrium and effective population sizes in Chinese Merino (Xinjiang type) sheep by genome-wide SNPs. *Genes Genomics* 39, 733–745. doi:10.1007/s13258-017-0539-2.

National Museum of Australia Merino sheep introduced. Available at: https://www.nma.gov.au/defining-moments/resources/merino-sheep-introduced [Accessed September 10, 2019].

Ovigen (2019). Raza OVINA Ojalada. *Ovigen*. Available at: http://ovigen.es/raza-ovina-ojalada/ [Accessed September 11, 2019].

Porter, V., Alderson, L., Hall, S. J. G., and Sponenberg, D. P. (2016). *Mason’s World Encyclopedia of Livestock Breeds and Breeding, 2 Volume Pack*. CABI.

Ren A, Li B, Jie HD, Chen L, Zhang B, Ao SM, Tan ZL, Zhou CS, Zhuzha BS, Chen WY, Hou SZ: Growth performance and meat quality in Tibetan sheep fed diets differing in type of forage. *Kafkas Univ Vet Fak Derg*, 2019. DOI: 10.9775/kvfd.2018.21144

Riva, J., Rizzi, R., Marelli, S., and Cavalchini, L. G. (2004). Body measurements in Bergamasca sheep. *Small Rumin. Res.* 55, 221–227. doi:10.1016/j.smallrumres.2003.12.010.

Sarti, F. M., Lasagna, E., Panella, F., Lebboroni, G., and Renieri, C. (2006). Wool quality in Gentile di Puglia sheep breed as measure of genetic integrity. *Ital. J. Anim. Sci.* 5, 371–376. doi:10.4081/ijas.2006.371.
