# Supplementary figures and images for: A Combined Multi-Cohort Approach Reveals Novel and Known Genome-Wide Selection Signatures for Wool Traits in Merino and Merino-Derived Sheep Breeds
Source: Front Genet. 2019 Oct 25;10:1025. doi: 10.3389/fgene.2019.01025 (PMC6824410; doi:10.3389/fgene.2019.01025)

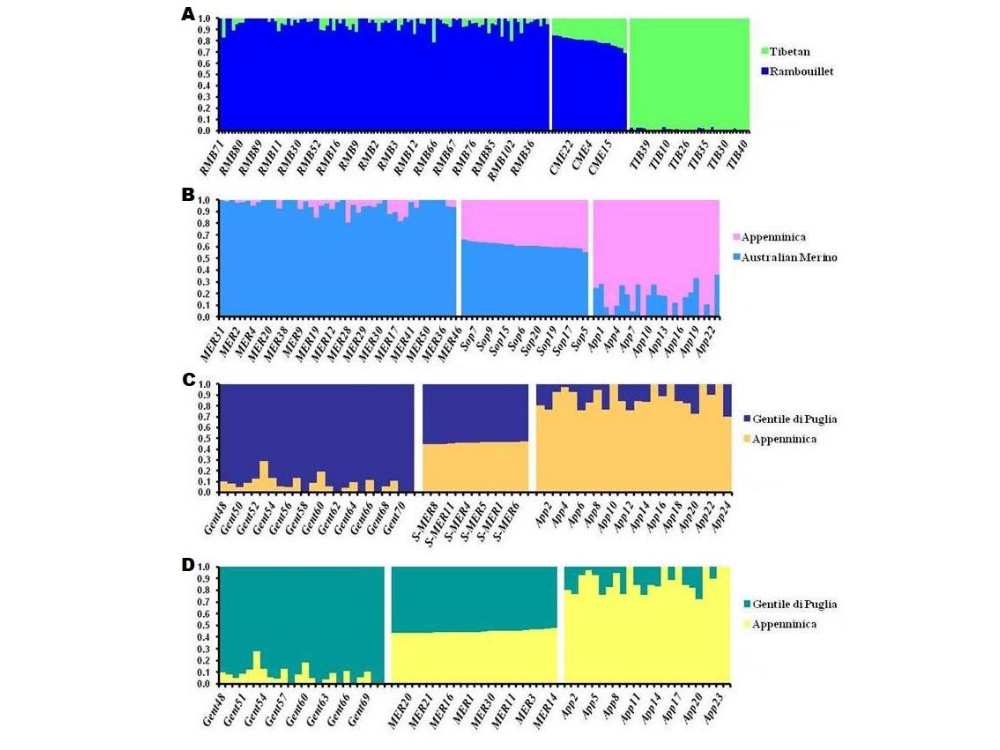

Supplement: Figure S1 — Individual proportions of global admixture for the four considered datasets. Dataset composition: (A) Rambouillet, Chinese Merino, Tibetan; (B) Australian Merino, Sopravissana, Appenninica; (C) Gentile di Puglia, Spanish Merino, Appenninica; (D) Gentile di Puglia, Australian Merino, Appenninica. To estimate admixture proportions of the four test breeds (A, Chinese Merino; B, Sopravissana; C, Spanish Merino; D, Australian Merino), each dataset was assumed to be arranged into two sub-populations. Color codes define the admixture proportions for each animal. Individual proportions of global admixture were averaged within breed to obtain a, the fraction of global admixture, adopted as parameter in the “local ancestry” analyses (see main text). [file Image_1.tif]
